# Involuntary Neuromuscular Coupling between the Thumb and Finger of Stroke Survivors during Dynamic Movement

**DOI:** 10.3389/fneur.2018.00084

**Published:** 2018-03-01

**Authors:** Christopher L. Jones, Derek G. Kamper

**Affiliations:** ^1^HD LifeSciences, Stoneham, MA, United States; ^2^UNC/NC State Joint Department of Biomedical Engineering, Rehabilitation Engineering Core, Raleigh, NC, United States

**Keywords:** exoskeleton, motor control, coupling, reflex, hand, robot, rehabilitation

## Abstract

Finger–thumb coordination is crucial to manual dexterity but remains incompletely understood, particularly following neurological injury such as stroke. While being controlled independently, the index finger and thumb especially must work in concert to perform a variety of tasks requiring lateral or palmar pinch. The impact of stroke on this functionally critical sensorimotor control during dynamic tasks has been largely unexplored. In this study, we explored finger–thumb coupling during close–open pinching motions in stroke survivors with chronic hemiparesis. Two types of perturbations were applied randomly to the index with a novel Cable-Actuated Finger Exoskeleton: a sudden joint acceleration stretching muscle groups of the index finger and a sudden increase in impedance in selected index finger joint(s). Electromyographic signals for specific thumb and index finger muscles, thumb tip trajectory, and index finger joint angles were recorded during each trial. Joint angle perturbations invoked reflex responses in the flexor digitorum superficialis (FDS), first dorsal interossei (FDI), and extensor digitorum communis muscles of the index finger and heteronymous reflex responses in flexor pollicis brevis of the thumb (*p* < 0.017). Phase of movement played a role as a faster peak reflex response was observed in FDI during opening than during closing (*p* < 0.002) and direction of perturbations resulted in shorter reflex times for FDS and FDI (*p* < 0.012) for extension perturbations. Surprisingly, when index finger joint impedance was suddenly increased, thumb tip movement was substantially increased, from 2 to 10 cm (*p* < 0.001). A greater effect was seen during the opening phase (*p* < 0.044). Thus, involuntary finger–thumb coupling was present during dynamic movement, with perturbation of the index finger impacting thumb activity. The degree of coupling modulated with the phase of motion. These findings reveal a potential mechanism for direct intervention to improve poststroke hand mobility and provide insight on prospective neurologically oriented therapies.

## Introduction

The dexterity of the digits of the hand is one of the hallmarks of human motor control and a central factor in the evolution of our species. The highly individuated movement enables complex and dynamic interaction with the environment, such as for manipulating tools and objects. Motion (1) and force (2) independence are especially great in the index finger and thumb, the two most functionally important digits.

Yet, coordination between these digits is critical for proper execution of a number of important tasks. During object manipulation through pinch, for example, the thumb and index fingertip forces must create equal and oppositely directed forces to prevent slip of the object. Alteration in the force created by one digit, such as might arise due to perturbations or changing conditions such as sweat, requires immediate compensation by the other digit.

The finger and thumb have multiple degrees-of-freedom (DOF) available which can be exploited to match the other digit’s movement; this redundancy, however, contributes substantially to variability in movement (3). By introducing coupling between these DOF, variability in motor output can be reduced (4), thereby improving performance of the digits in a coordinated task. Accordingly, research has shown evidence for neural coupling between neuromuscular units for different digits in the human hand. For example, EMG–EMG coherence was observed between pairs of finger and thumb muscles during a pinching task (5) and during a three-digit grasping paradigm (6).

In stroke survivors, unfortunately, coordination between finger and thumb may be disrupted. For example, we have observed strong coupling between thumb and finger flexors when stretch is applied to the nominally passive finger flexors (7). We have also seen this aberrational finger flexor-thumb flexor coupling during voluntary isometric task performance (7).

Study of finger–thumb coupling during dynamic tasks, however, has been limited. While perturbation techniques are often used to study motor control in the arm (8), this methodology is more challenging in the hand due to the many DOF present within a relatively small volume. Cole and Abbs examined response to an extension perturbation of the thumb in neurologically intact individuals (9, 10), but thumb motion was limited to a single joint and perturbations were applied to only one thumb joint. Schettino et al. recently examined perturbation of the index finger during a reach-to-grasp task, but kinetic perturbations only were applied and muscle activation patterns were not addressed (11).

Even fewer such studies have been performed with stroke survivors. Thus, for this study, we examined thumb-finger coupling during a natural dynamic movement in stroke survivors. Using a novel actuated finger exoskeleton, we introduced precise perturbations to the index finger during a voluntary palmar pinch-open task. First, we investigated possible reflex coupling at the spinal level by applying rapid rotation of the metacarpophalangeal (MCP) joint to evoke a stretch reflex in the finger muscles. We hypothesized that this would elicit heteronymous reflexes in the unperturbed thumb motoneurons with a similar delay. We further hypothesized that this coupling would be stronger when perturbations were applied during the closing phase of a pinch movement than when applied during digit opening. Next, we examined possible hierarchical control during the pinching task by perturbing the index finger trajectory. We hypothesized that perturbations which altered the index finger trajectory would lead to corresponding alterations in the pathway of the thumb to reduce task error. Furthermore, we expected the effect on thumb movement to be greater when the index finger was perturbed during the closing phase of pinch when coupling of movement was most critical.

## Materials and Methods

**Figure 1:**
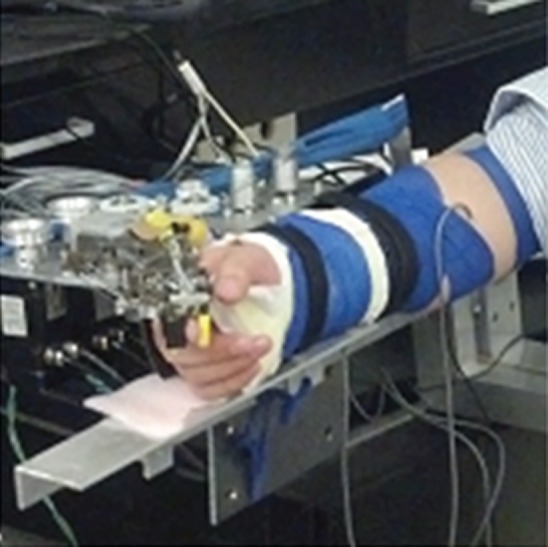
Finger connected to Cable-Actuated Finger Exoskeleton device for performing the experiments. Motors are located off the splinted hand and wrist such that they can be supported by an external structure, the TA-WREX (21, 22).

The Cable-Actuated Finger Exoskeleton (CAFE) (12, 13) was employed to perturb joints of the index finger. This rigid exoskeleton structure has joints that are aligned with the flexion/extension axis of each of the three index finger joints: metacarpophalangeal (MCP), proximal interphalangeal (PIP), and distal interphalangeal (DIP). The structure runs along the radial side of the finger and couples to the finger through bars contacting the dorsal and palmar sides of each finger segment. Rotation of the exoskeleton joint, thus, produces equivalent rotation of the anatomical joint. Cables running from electric servomotors located proximal to the wrist connect to gears located at each joint (Figure 1). Winding of the cable about a spool connected to the motor thereby produces joint rotation. One motor produces flexion and another produces extension at each joint, for a total of six motors. The cables run through a series of pulleys before terminating at the appropriate joint. By placing the gearing directly at the joint, the relative influence of cable force at a joint other than the targeted joint is reduced. Further compensation is achieved through the controller. Precise, independent control of each joint of the index finger can be achieved over a wide range of velocities and torques.

### Participants

A convenience sample of eight adult individuals with chronic hemiparesis resulting from a single stroke incurred at least 6 months prior, with a mean (±SD) age of 63 (±9) years, participated in the study. Subjects were selected based on having moderate hand impairment as characterized by a rating of Stage of Hand 4 or 5 on the Chedoke-McMaster Stroke Assessment (14). Subjects at these levels typically exhibit substantial gross finger extension, but have difficulty producing individuated finger movements. A significant spastic reflex in the finger flexors has been detected in stroke survivors with this level of impairment (15). The group consisted of two females and six males. All participants were right-hand dominant. This investigation was conducted at the Rehabilitation Institute of Chicago and all participants provided written consent in accordance with processes approved by the Northwestern University Institutional Review Board.

### Protocol

Each subject participated in two sessions. During the initial session, we captured the kinematics of the subject’s natural pinching motion while the wrist was held in a fixed, neutral posture. Beginning with the finger and thumb positioned in an open-handed posture by the device, subjects were asked to create a palmar pinching motion with the index finger and thumb (closing phase). Once the thumb and index finger made approximate contact, the subject was instructed to immediately begin to open the digits to return them to the original posture (opening phase). Precise contact position and movement duration was subject-specific to allow completion of movement and a naturally paced trajectory, although participants were required to complete the full close–open motion within 2 s for a minimum frequency of 0.5 Hz. All movements were initiated with audible cues to first prepare to move and then to initiate movement. The kinematics of the index finger during the movement were recorded using an external camera system (Optotrak, 3020, 3010, Northern Digital, Inc., Waterloo, ON, Canada) employing infrared markers at each of the finger joints (MCP, PIP, DIP) and fingertip. Marker locations were sampled at a rate of 100 Hz. We subsequently used these data to compute the joint angles that served as the desired motion trajectories in the second session.

During the second session, subjects participated in two sets of experimental conditions, each consisting of a within-subject repeated measures experimental design to examine finger–thumb interactions during voluntary movement. Digit kinematics and muscle activity were measured for both sets of experimental conditions. Thumb tip location was captured with the Optotrak camera system, while index finger joint angles were recorded by the CAFE at 1 kHz. Activation of specified finger and thumb muscles, selected for their participation in finger–thumb pinch (16–20) and accessibility for electrode placement, were recorded with EMG electrodes. Surface electrodes (Delsys, Inc., Boston, MA, USA) were placed over flexor digitorum superficialis (FDS), extensor digitorum communis (EDC), and first dorsal interossei (FDI) of the index finger and over flexor pollicis brevis (FPB) and abductor pollicis brevis (APB) of the thumb. EMG signals were sampled at 1 kHz. Data collection is synchronized at time of collection between the Optotrak and EMG DAQ simultaneously *via* a shared electrical signal.

With the subject seated comfortably, we coupled their index finger to the CAFE device. We then splinted the subject’s wrist and forearm to a platform of an external device (TA-WREX) (21, 22) to support the weight of the arm and the exoskeleton motors and fix wrist flexion/extension and abduction/adduction.

#### Joint Angular Perturbation

To examine index finger–thumb reflexive coupling during goal-directed palmar pinching, we instructed subjects to create the same pinching motion as they did during the first session. In this session, however, the CAFE moved according to the joint angle trajectories recorded during the prior session. Subjects performed isokinetic movements with the index finger and were instructed to push against the device in the direction of motion during movement, resulting in an average muscle force generation of roughly 10% of maximum voluntary contraction (MVC) throughout both the closing and opening phases. This baseline muscle activation increases the probability of generating a reflex in response to applied muscle stretch (23). To determine the MVC, a series of three alternating flexion and extension contractions are performed prior to trials, the peak EMG values of which are compared to the peak EMG envelope following reflex response; the greatest of which are taken as the MVC value for the respective muscle.

**Figure 2:**
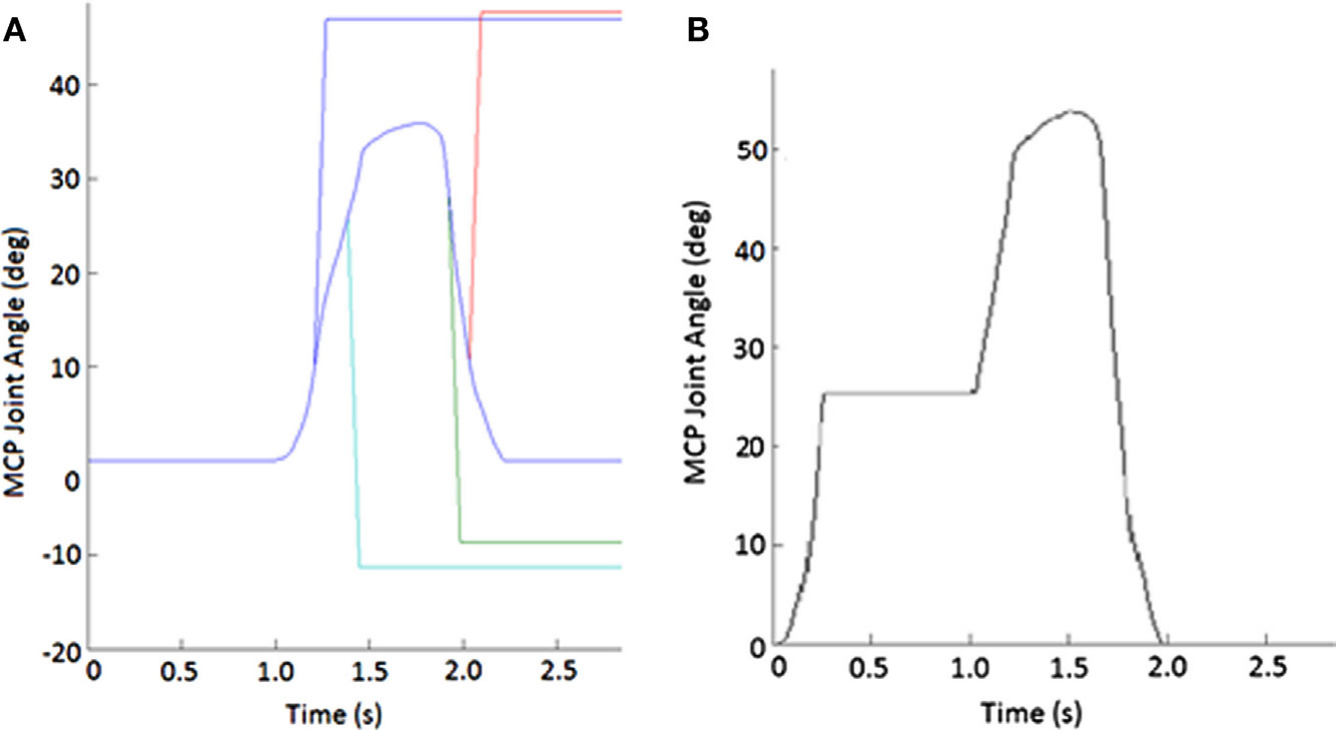
Example of perturbations. **(A)** Displacement applied to metacarpophalangeal (MCP) joint to create stretch of finger muscles. Blue line shows unperturbed subject-specific trajectory while the other lines show flexion (increasing angle) or extension (decreasing joint angle) or extension imposed during either the closing or opening phase of movement. **(B)** Impedance of MCP joint suddenly increased for 750 ms during the closing phase of the pinching task.

During random trials, position-controlled angular perturbations of approximately 40° at 600°/s were applied to the MCP joint of the index finger to elicit a stretch reflex in certain index finger muscles. These perturbations were applied roughly halfway through either the opening or closing movement phase (±10° variation), in either the flexion (stretching MCP extensors) or extension (stretching MCP flexors) direction (Figure 2A). Ten trials were performed for each phase-direction perturbation condition (closing-extension, closing-flexion, opening-extension, and opening-flexion), along with 20 no-perturbation control trials, for a total of 60 trials. A series of at least 10 control trials were presented before any perturbation was introduced to allow the participant to become familiarized with the system. Additionally, we exposed subjects to the movement of the device to test the fit and the accuracy of the movement profile, as well as to build comfort with the system, prior to the beginning of the first control trials.

#### Joint Impedance Perturbation

In the second set of trials, we used the CAFE to disrupt the movement of the finger during natural palmar pinching motions. Subjects created the same close–open pinching movement as in the previous experiment. However, in this experiment, the exoskeleton minimizes contact force between itself and the user, thus reducing the muscle activation required to move and not also preventing the exoskeleton from assisting in the movement.

During certain trials, the CAFE applied an impedance perturbation to the MCP or PIP + DIP (perturbed together) joints of the index finger. The impedance perturbation consisted of an abrupt transition to a very stiff joint, essentially locking movement of the joint(s) of the device for 750 ms (Figure 2B). In this manner, we could alter the index finger trajectory without displacing the joints (which could evoke a stretch reflex). Perturbations were applied approximately halfway through each phase of movement (±10° variation). Thus, there were four phase-joint perturbation combinations: MCP perturbation during closing (closing-MCP), IP perturbation during closing (closing-IP), MCP perturbation during opening (opening-MCP), and IP perturbation during opening (opening-IP). Each subject performed 10 trials of each perturbation condition with 20 no-perturbation control trials, presented in random order, for a total of 60 trials. A series of at least 10 control trials was presented before any perturbation was introduced, and rest was provided as needed.

### Data Analysis

EMG signals were analyzed to quantify the reflex response for the experiment involving rapid muscle stretch. Thumb motion data from the Optotrack were the primary outcome measure for the experiment involving the sudden change in exoskeleton joint impedance.

#### Stretch Reflex

To find the shorter-latency reflex responses to the perturbations, we examined the EMG envelope during the 150-ms window following the onset of perturbation. This time span captures the reflexive, but not voluntary muscle activation in response to perturbation. EMG of each muscle was rectified and then low-pass filtered forwards and backwards through a fifth-order Butterworth filter with a cutoff frequency of 40 Hz to create the EMG envelope. This envelope was then normalized by the maximum envelope value across all trials and the initial MVCs for the corresponding muscle and subject.

From these absolute measures, perturbed and unperturbed EMG, we created two outcome measures: A-EMG, the absolute value of each normalized EMG signal and D-EMG, the difference between the EMG during the perturbed trial and the unperturbed trials. In order to examine whether the stretch produced reflex activity, we compared peak A-EMG with and without perturbation for each muscle by employing multiple analysis of covariance (MANCOVA). Due to violations in assumptions of normality, we used the non-parametric Mann–Whitney *U* test to look at the impact of movement phase (closing/opening) and stretch direction (extension/flexion) on D-EMG. We examined the impact of phase and direction on time to peak reflex EMG response using MANCOVA. Individual *post hoc* ANOVA are performed to quantify individual effects where appropriate.

#### Impedance Perturbation

For the impedance perturbation experiment, we examined thumb tip kinematics, recorded with the Optotrak system. We first examined the aperture during the movement and then focused on the time window covering the period from the start of index finger perturbation until the time at which the unperturbed phase ended. Specifically, we computed the normal distance between fingertip and thumb tip Optotrak markers, computing the total aperture during movement.

We then isolated the thumb movement from the exoskeleton-controlled finger movement by computing the Euclidian norm of the thumb tip position, zeroed at the angle and the time of perturbation (positive indicates closing and negative indicates opening). We then compared the thumb trajectory for each individual trial to the average trajectory of the unperturbed trials for the same subject by computing the root mean squared error (RMSE) between the two. We employed ANOVA to examine the effects of condition (perturbed/unperturbed), movement phase (closing/opening), and joint (MCP/IP) on RMSE.

## Results

Subjects performed pinching movements in the CAFE as instructed for both the reflex and impedance experiments. All subjects completed both sets of experiments.

### Stretch Perturbation

**Figure 3:**
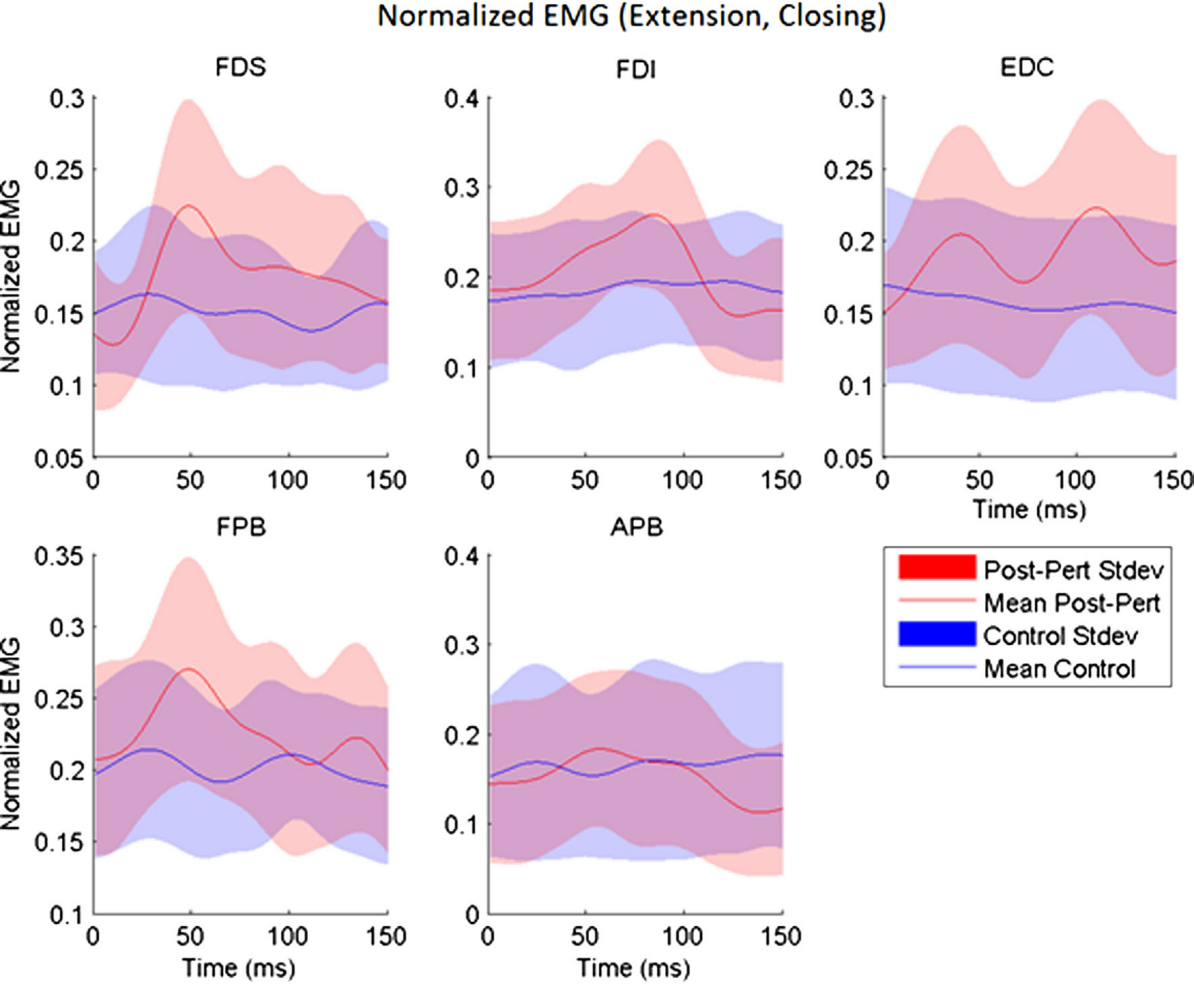
Across-subjects mean EMG envelopes for each muscle following extension perturbation during the closing phase. Unperturbed (blue) and perturbed (red) conditions are shown with their across-subjects mean values (line) and associated SDs (shaded).

While subjects made active, volitional pinching movements, stretch perturbations produced strong reflex activity. Post-perturbation peak EMG was greater than the time-matched EMG for unperturbed trials for every muscle (*p* < 0.017) with the exception of APB. A-EMG (absolute magnitude) following perturbation was typically greater than in the unperturbed case by 10–15% of MVC. Thus, stretch of a given set of finger muscles produced reflex responses in all observed index finger muscles, as well as in the thumb flexor FPB (Figure 3). However, no such reflex response was observed in APB (Figure 3).

**Figure 4:**
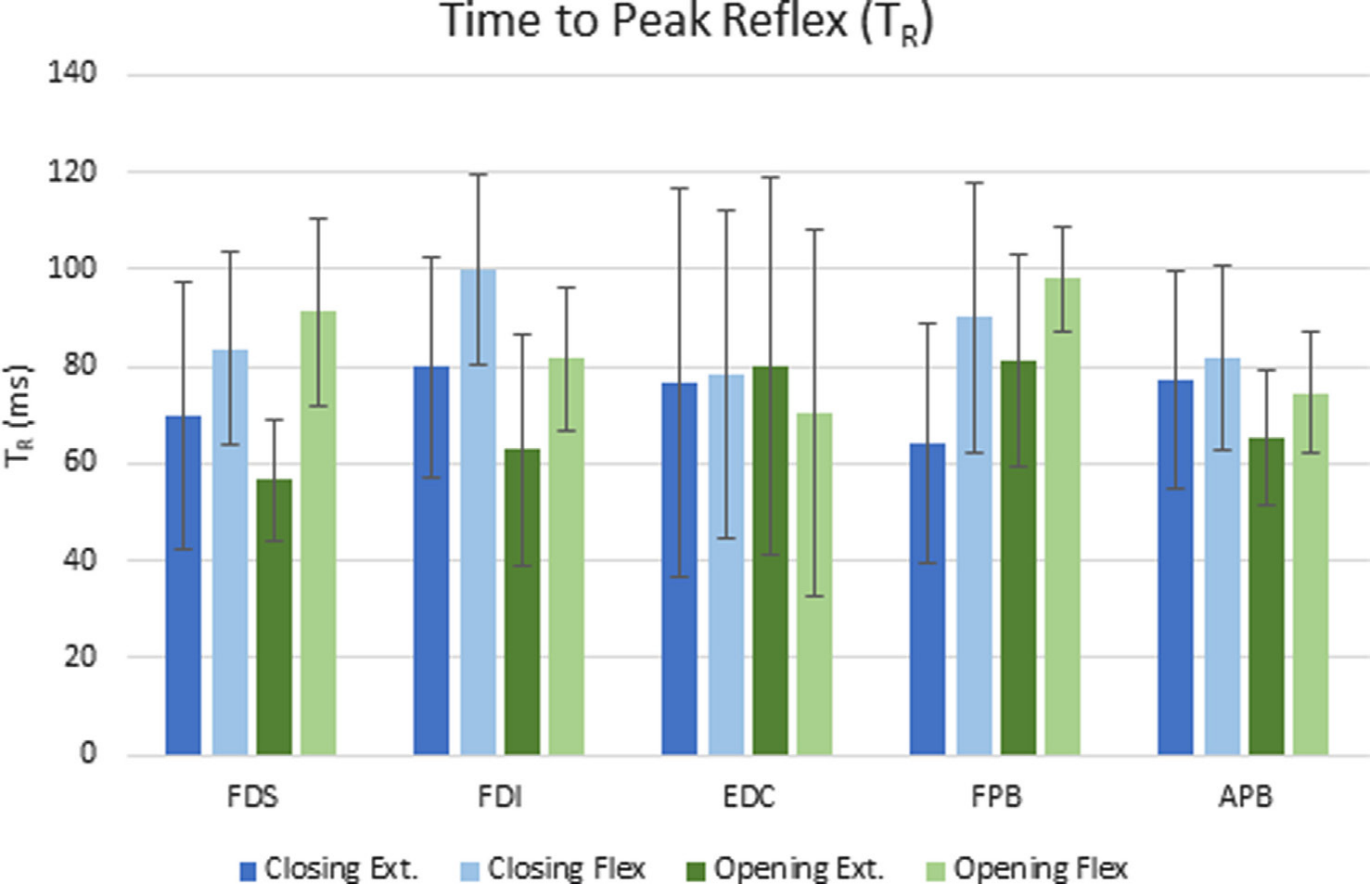
Mean elapsed time (ms) to peak reflex response (TR) for each condition across subjects. Error bars indicate ±1 SD.

The delay between the initiation of the perturbation and the time to peak reflex EMG (TR) was significantly dependent upon both movement phase (closing or opening) and perturbation direction (flexion or extension), but the size of the reflex response (D-EMG) did not vary significantly with either. Moving the MCP into extension (thereby lengthening the finger flexor muscles), resulted in a shorter time to peak reflex for both FDS and FDI (*p* < 0.012). Furthermore, TR for FDI was also significantly affected by phase of the movement (*p* < 0.002) such that the delay was shorter during the opening portion of the movement than during the closing portion. The mean delay for FDS and FPB was very similar for a stretch perturbation of FDS during closing, but the FPB peak response was delayed by 25 ms, on average, with respect to the FDS peak reflex response during opening (Figure 4).

### Impedance Perturbation

**Figure 5:**
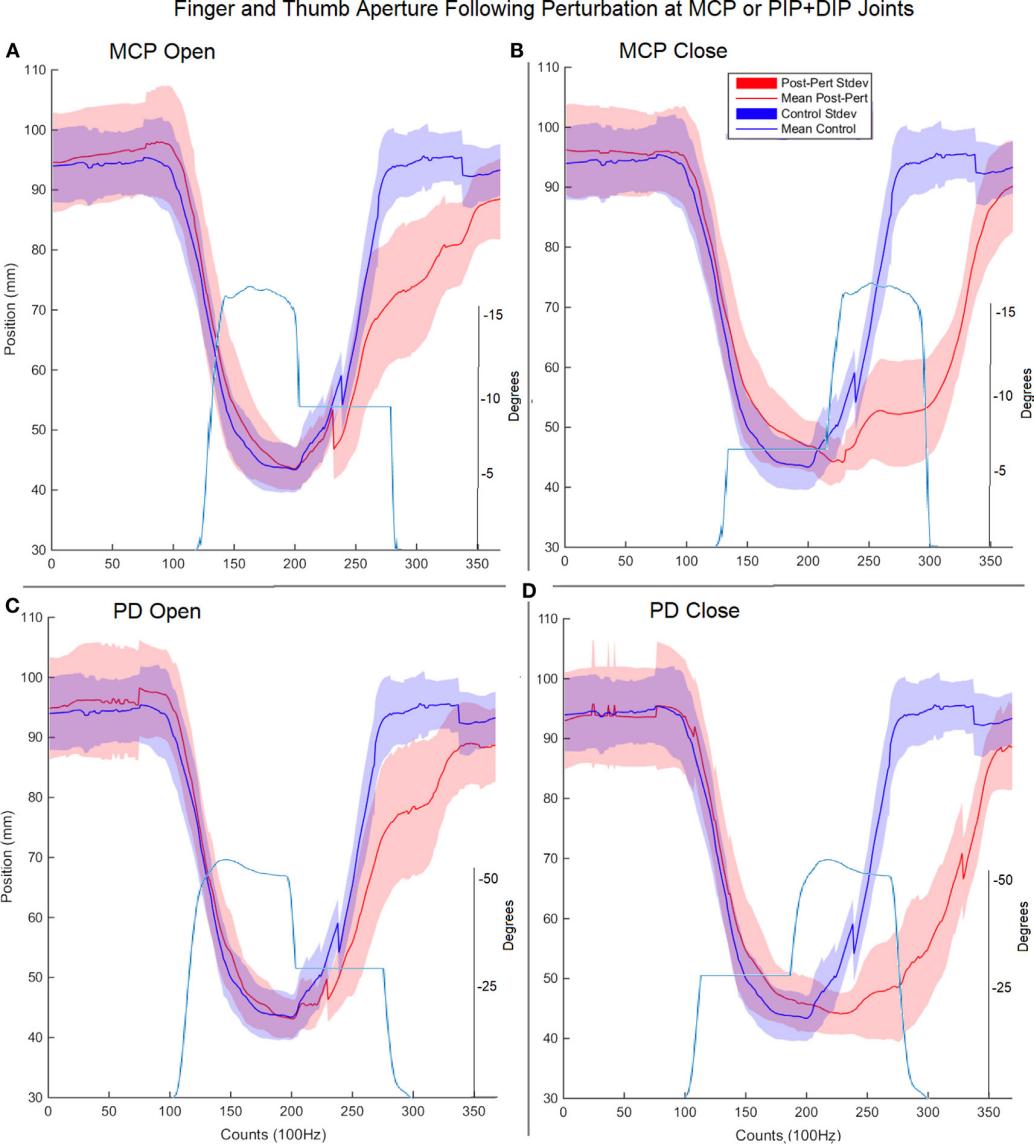
Finger and thumb aperture during perturbed (red) and unperturbed (blue) trials following perturbations. Each perturbation condition is shown, **(A)** metacarpophalangeal (MCP) perturbation during opening, **(B)** MCP perturbation during closing, **(C)** proximal interphalangeal (PIP)/distal interphalangeal (DIP) joint during opening, **(D)** PIP/DIP during closing. Examples of joint angle trajectories with perturbations are provided for reference; note, flat region indicates joint-locked perturbation.

Surprisingly, for each type of impedance perturbation (MCP/IP, closing/opening), the aperture remained relatively unchanged (Figure 5). This is evident during the onset of each perturbation where the finger is delayed, indicating the thumb movement is accelerated in the absence of finger movement.

**Figure 6:**
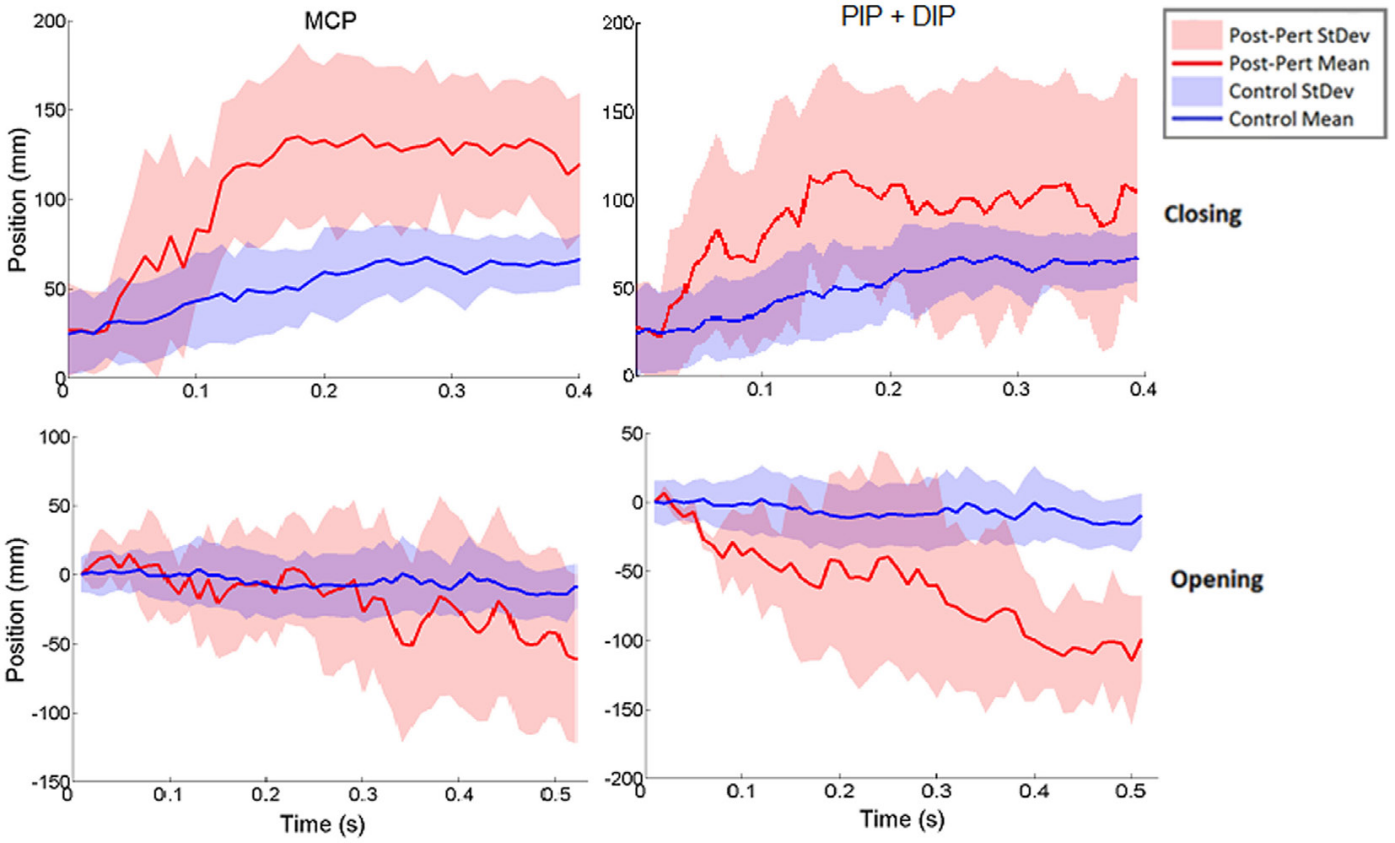
Norm of 3D thumb tip displacement vector following metacarpophalangeal (MCP) (left column) or IP (right column) joint-locked perturbation during closing (top) or opening (bottom). Movement is shown for the period of time from initiation of perturbation to end of movement phase for the unperturbed trials. Mean perturbed (red line) and unperturbed (blue line) trajectories are shown with associated SDs (shaded regions). *Y*-axis zeroed to the angle of perturbation such that negative angles are in closing and positive angles are in opening directions.

**Figure 7:**
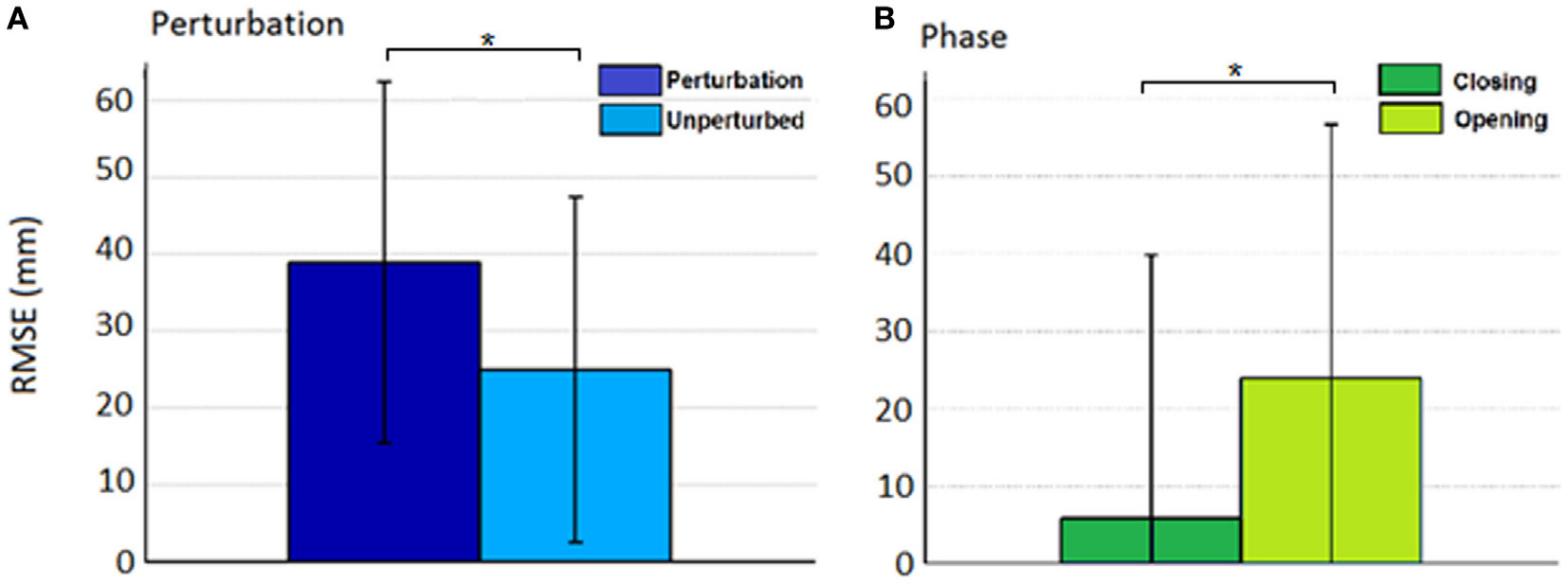
Root mean squared error (RMSE) for the effect of **(A)** condition (perturbed/unperturbed) and **(B)** phase (closing/opening).

Thus, rather than stopping or slowing to match the checked movement of specific index finger joints, thumb movement increased beyond previous levels (Figure 6). ANOVA results revealed that the RMSE in thumb trajectory from the mean unperturbed trajectory was significantly impacted by the perturbation (*p* < 0.001), with greater RMSE during perturbed trials (Figure 7A).

The increased thumb movement occurred in the intended direction of movement. Hence, during closing, thumb flexion after index finger perturbation became greater, more closely matching what would be expected in unimpaired individuals performing this movement, particularly at the thumb MCP joint. Even more startling, a large increase in thumb extension was observed for perturbations applied to the index finger during the opening phase. Thus, RMSE was significantly affected by phase of movement (*p* < 0.044), with greater RMSE during opening (Figure 7B). While greater mean thumb displacements were observed for perturbation of the MCP joint during closing and the IP joints during opening, there was no significant effect of the joint(s) perturbed (finger MCP or IP) on thumb movement.

## Discussion

Using a novel finger exoskeleton, we were able to assess involuntary coupling present between the thumb and index finger during a dynamic movement in stroke survivors. Kinematic and EMG data revealed strong, perturbation-dependent interactions between index finger and thumb muscles in the stroke survivors.

### Reflex Response

Imposed stretch perturbations of the index finger muscles during the dynamic task evoked significant reflex EMG activity in all of the measured index finger muscles. In nominally passive stroke survivors (those who are not actively utilizing muscles), we have observed that a similar rapid stretch of the finger flexors, such as FDS, likewise produces a significant stretch reflex in the stretched muscles (24). This study shows that this behavior is also evident during voluntary movement in stroke survivors, as has been described for the elbow (25, 26). The observed reflex behavior in shortening muscles, such as EDC during an extension perturbation, is reminiscent of the occasional reflex response observed in EDC during stretch of the spastic flexors in passive stroke survivors (24). It should be noted that the delay to the peak EDC reflex during extension was longer than that for FDS, thereby suggesting a longer reflex loop.

Stretch of EDC during the flexion perturbation also resulted in reflex generation. This contrasts to the case in passive stroke survivors, within whom stretch of finger extensors such as EDC generally fails to produce any reflex response (15). The EDC stretch produced a reflex response present in the finger flexors as well. Reflex activity in the non-stretched muscles may arise from loss of reciprocal inhibition or even a transition to reciprocal excitation following the stroke (27, 28).

In support of our hypothesis, we also observed reflex coupling between certain finger and thumb muscles. Thus, stretch of finger muscles produced reflex responses in a non-stretched thumb muscle, FPB. We previously observed this phenomenon in passive stroke survivors (29), but this is the first time we have been able to verify that these coupled reflexes can be evoked during voluntary movement. Intriguingly, significant reflex behavior was not induced in all thumb muscles, but rather in FPB and not in APB. Thus, it appears that coupling may be greater between thumb and finger flexors, as we observed in the passive condition (29). It should be noted that rapid stretch of thumb muscles in passive stroke survivors failed to elicit a spastic stretch reflex (30). Limited APB reflex may also be attributed to a reduction in heterogeneous extrinsic–intrinsic connections (7). FDS, FDI, and FPB reflex timing follow the same temporal pattern of reflex activation with peak EMG occurring 19–25 ms earlier for extension perturbations, as compared with flexion perturbations. It should be noted that thumb movement was controlled voluntarily throughout the task and so may have varied. As one head of FDI originates on the thumb metacarpal, thumb movement may have influenced FDI length and thus excitability, resulting in increased variability between trials and differing strategies between subjects.

The magnitude of the heteronymous reflex activity observed in the thumb muscles due to stretch of the index finger muscles did not vary significantly with movement phase. However, while the time to peak reflex response was very similar for FDS and FPB during closing, it was much longer for FPB during opening, when less coordination was required between the thumb and index finger. Thus, modulation of the finger–thumb coupling may occur with movement phase, although it may be partially impaired in stroke survivors. Indeed, the interaction of phase and direction was non-significant for every muscle. This absence of an effect may suggest the loss of capacity for modulating index finger and thumb neurological coupling specific to the motor control task. This is consistent with previous findings of unmodulated hyperreflexia across static postures of the wrist (31) where, despite changing posture of the wrist, reflex gains remained unmodulated, as well as a general deficit in modulation of EMG patterns in the finger and thumb (32, 33). By contrast, phase-dependent reflex modulation has been reported in a previous study examining cyclical arm movements (cycling) in neurologically intact individuals (34).

### Impedance Perturbation

The response of thumb tip motion to imposed disruption of index finger movement was especially intriguing. Contrary to our hypothesis, impeding finger movement led to increased thumb movement. The thumb is observed to maintain the anticipated aperture, indicating an accelerated trajectory. This movement is beyond what was achieved without any perturbations, flexing further during closing and extending further during opening following perturbation. During the closing phase perturbed trials, thumb motion showed acceleration just prior to reaching the unperturbed point of contact, thereby suggesting that the thumb was indeed responding to an imposed deficit in the index finger by moving further than normal to meet the finger. This movement may have been encouraged by stabilizing the index finger *via* increased joint impedance. Alternatively, greater thumb movement may have arisen as the result of increased somatosensory feedback arising from perturbation contact forces in the index finger.

The most striking result was the increase in thumb extension during the opening phase for the perturbed trials. Stroke survivors typically have difficulty in creating active thumb extension (34). This was evident in our stroke survivors who typically generated less than 2 cm of thumb extension during the unperturbed trials. When the index finger impedance was suddenly increased, however, mean thumb extension increased to over 10 cm, an amount roughly equivalent to what would be expected in neurologically intact subjects. The mechanisms behind this improvement are unclear. One possibility is that the perturbation leads to increased involuntary activation of all digit extensor muscles.

### Coupling

The presence of a coupled response between the index finger and thumb affirms previous findings during coordinated rapid grasping tasks in neurologically intact individuals (9, 10, 35) and indicates a preservation of coupling following stroke. Thumb kinematic responses to index finger perturbation align with previously demonstrated influence over thumb kinematics during healthy grasp (36). This outcome supports the notion of preservation of components of motor control following stroke, including coupling between digits (37, 38). The presence of phase changes indicates there is some preservation of modulation of index finger–thumb coupling, in contrast to findings following reflex-inducing perturbations. The modulation effects are present across larger temporal scales (0.5–0.75 s).

The remarkable improvement of thumb movement in response to impedance of index finger movement may arise as the result of excitatory or inhibitory coupling between index finger and thumb muscles. During the opening phase, impedance to finger movement could give rise to increased activation of finger extensor muscles which, in the presence of coupling to the thumb, could create reciprocal excitation of thumb extensors and/or reciprocal inhibition of thumb flexors; both of these would result in improved movement of the thumb. However, due to the use of surface electrodes, extensor EMG data were not available for the thumb in this study; efforts should be made to include thumb extensor EMG in future investigations.

One such route of coupling may follow the reticulospinal pathway, which has been demonstrated to integrate somatosensory feedback into motor control (39). Inhibition through such a pathway may improve movements in the presence of large somatosensory stimuli. In this way, somatosensory stimulation of the index finger may provide a pathway for intervention following stroke to promote thumb movement. Similar targeted haptic feedback has been shown to improve motor control in the arm (40–42).

### Potential Limitations

These experiments only examined reflexes during part of the movement: midway through the closing and opening phases of movement. Further experiments examining reflex modulation across the range of postures during the movement would better inform the extent of modulation and contributions of reflex activity to motor control during pinch.

Sample size was limited. Part of the goal of this study was to ensure feasibility of use of the CAFE with stroke survivors. Further exploration of the observed increase in thumb extension resulting from index finger perturbation in more subjects is warranted. Future work in conjunction with a thumb exoskeleton or other enhanced thumb kinematic measures will enable increased insight into thumb muscle behavior.

Additionally, reflex modulation may have been impacted by contact with the exoskeleton. Somatosensory cutaneous afferents have been shown to contribute to neuromodulation at the spinal cord (43). Contact with the exoskeleton was designed to be constant throughout the flexion or extension phase as subjects were instructed to maintain a specific voluntary activation level. Coupling of interaction between finger and thumb extensor muscles could not be examined as thumb extensor activity was not monitored.

## Conclusion

While the index finger and thumb are capable of highly individuated movements, they also exhibit substantial coupling in tasks requiring finger–thumb coordination. These results suggest that coupling is highly evident in stroke survivors and appears to maintain behavior appropriate to task despite the underlying hand impairment.

In particular, the thumb clearly experienced a coupled response during the dynamic pinching task following index finger perturbation. Intriguingly, sudden arrest of index finger extension led to a profound increase in active thumb extension, far beyond what was generated without the perturbation. Further research is needed to validate and explore this finding, but the potential significance for rehabilitation is great.

## Ethics Statement

This investigation was conducted at the Rehabilitation Institute of Chicago (RIC) and all participants provided written consent in accordance with processes approved by the Northwestern University Institutional Review Board.

## Author Contributions

This work was conceived, conducted, analyzed, and authored by CJ and DK.

## Conflict of Interest Statement

The authors declare that the research was conducted in the absence of any commercial or financial relationships that could be construed as a potential conflict of interest.

## References

[1] Schieber MH Santello M Hand function: peripheral and central constraints on performance. J Appl Physiol (2004) 96(6):2293–300. 10.1152/japplphysiol.01063.2003 15133016

[2] Gao F Latash ML Zatsiorsky VM. Internal forces during object manipulation. Exp Brain Res (2005) 165(1):69–83. 10.1007/s00221-005-2282-1 15912369 PMC2847586

[3] Santello M Flanders M Soechting JF. Patterns of hand motion during grasping and the influence of sensory guidance. J Neurosci (2002) 22(4):1426–35. 11850469 10.1523/JNEUROSCI.22-04-01426.2002 PMC6757566

[4] Santello M Kinematic synergies for the control of hand shape. Arch Ital Biol (2002) 140(3):221–8. 12173525

[5] Kilner JM Alonso-Alonso M Fisher R Lemon RN. Modulation of synchrony between single motor units during precision grip tasks in humans. J Physiol (2002) 541(Pt 3):937–48. 10.1113/jphysiol.2001.013305 12068052 PMC2290366

[6] Poston B Enoka JA Enoka RM. Endpoint accuracy for a small and a large hand muscle in young and old adults during rapid, goal-directed isometric contractions. Exp Brain Res (2008) 187(3):373–85. 10.1007/s00221-008-1309-9 18288474

[7] Kamper DG Fischer HC Conrad MO Towles JD Rymer WZ Triandafilou KM. Finger-thumb coupling contributes to exaggerated thumb flexion in stroke survivors. J Neurophysiol (2014) 111(12):2665–74. 10.1152/jn.00413.2013 24671534 PMC6442660

[8] Lacquaniti F Borghese NA Carrozzo M. Transient reversal of the stretch reflex in human arm muscles. J Neurophysiol (1991) 66(3):939–54. 10.1152/jn.1991.66.3.939 1753296

[9] Cole KJ Abbs JH. Kinematic and electromyographic responses to perturbation of a rapid grasp. J Neurophysiol (1987) 57(5):1498–510. 10.1152/jn.1987.57.5.1498 3585477

[10] Cole KJ Gracco VL Abbs JH. Autogenic and nonautogenic sensorimotor actions in the control of multiarticulate hand movements. Exp Brain Res (1984) 56(3):582–5. 10.1007/BF00238001 6499984

[11] Schettino LF Adamovich SV Tunik E. Coordination of pincer grasp and transport after mechanical perturbation of the index finger. J Neurophysiol (2017) 117(6):2292–7. 10.1152/jn.00642.2016 28331008 PMC5461664

[12] Jones CL Wang F Osswald C Kang X Sarkar N Kamper DG Control and kinematic performance analysis of an actuated finger exoskeleton for hand rehabilitation following stroke. IEEE/RAS-EMBS BioRob. Tokyo, Japan (2010). p. 282–7.

[13] Jones CL Wang F Morrison R Sarkar N Kamper DG Design and development of the cable actuated finger exoskeleton for hand rehabilitation following stroke. IEEE Trans Mechatron (2014) 19(1):131–40. 10.1109/TMECH.2012.2224359 PMC6419777 30880898

[14] Gowland C VanHullenaar S Torresin W Moreland J Vanspall B Barrecca S Chedoke-McMaster Stroke Assessment: Development, Validation and Administration Manual. Hamilton, Canada: Chedoke-McMaster Hospitals and McMaster University (1995).

[15] Kamper DG Fischer HC Cruz EG Rymer WZ. Weakness is the primary contributor to finger impairment in chronic stroke. Arch Phys Med Rehabil (2006) 87(9):1262–9. 10.1016/j.apmr.2006.05.013 16935065

[16] Maier MA Hepp-Reymond MC. EMG activation patterns during force production in precision grip. II. Muscular synergies in the spatial and temporal domain. Exp Brain Res (1995) 103(1):123–36. 10.1007/BF00241970 7615028

[17] Maier MA Hepp-Reymond MC. EMG activation patterns during force production in precision grip. I. Contribution of 15 finger muscles to isometric force. Exp Brain Res (1995) 103(1):108–22. 10.1007/BF00241969 7615027

[18] Danion F Gallea C. The relation between force magnitude, force steadiness, and muscle co-contraction in the thumb during precision grip. Neurosci Lett (2004) 368(2):176–80. 10.1016/j.neulet.2004.07.006 15351444

[19] Goetz TJ Costa JA Slobogean G Patel S Mulpuri K Travlos A. Contribution of flexor pollicis longus to pinch strength: an in vivo study. J Hand Surg Am (2012) 37(11):2304–9. 10.1016/j.jhsa.2012.07.027 23101527

[20] Gagne M Schneider C. Dynamic changes in corticospinal control of precision grip during wrist movements. Brain Res (2007) 1164:32–43. 10.1016/j.brainres.2007.06.014 17632089

[21] Iwamuro BT Cruz EG Connelly LL Fischer HC Kamper DG. Effect of a gravity-compensating orthosis on reaching after stroke: evaluation of the Therapy Assistant WREX. Arch Phys Med Rehabil (2008) 89(11):2121–8. 10.1016/j.apmr.2008.04.022 18996241

[22] Housman SJ Scott KM Reinkensmeyer DJ. A randomized controlled trial of gravity-supported, computer-enhanced arm exercise for individuals with severe hemiparesis. Neurorehabil Neural Repair (2009) 23(5):505–14. 10.1177/1545968308331148 19237734

[23] Crago PE Houk JC Hasan Z. Regulatory actions of human stretch reflex. J Neurophysiol (1976) 39(5):925–35. 10.1152/jn.1976.39.5.925 978238

[24] Kamper DG Rymer WZ. Quantitative features of the stretch response of extrinsic finger muscles in hemiparetic stroke. Muscle Nerve (2000) 23(6):954–61. 10.1002/(SICI)1097-4598(200006)23:6<954::AID-MUS17>3.0.CO;2-0 10842274

[25] Mizrahi EM Angel RW Impairment of voluntary movement by spasticity. Ann Neurol (1979) 5(6):594–5. 10.1002/ana.410050620 475355

[26] Knutsson E Martensson A Gransberg L. Influences of muscle stretch reflexes on voluntary, velocity-controlled movements in spastic paraparesis. Brain (1997) 120(Pt 9):1621–33. 10.1093/brain/120.9.1621 9313644

[27] Nielsen JB Petersen NT Crone C Sinkjaer T. Stretch reflex regulation in healthy subjects and patients with spasticity. Neuromodulation (2005) 8(1):49–57. 10.1111/j.1094-7159.2005.05220.x 22151383

[28] Crone C Petersen NT Gimenéz-Roldán S Lungholt B Nyborg K Nielsen JB. Reduced reciprocal inhibition is seen only in spastic limbs in patients with neurolathyrism. Exp Brain Res (2007) 181(1):193–7. 10.1007/s00221-007-0993-1 17571255

[29] Triandafilou KM Fischer HC Towles JD Kamper DG Rymer WZ. Diminished capacity to modulate motor activation patterns according to task contributes to thumb deficits following stroke. J Neurophysiol (2011) 106(4):1644–51. 10.1152/jn.00936.2010 21753022

[30] Towles JD Kamper DG Rymer WZ. Lack of hypertonia in thumb muscles after stroke. J Neurophysiol (2010) 104(4):2139–46. 10.1152/jn.00423.2009 20668270 PMC2957448

[31] Meskers CG Schouten AC de Groot JH de Vlugt E van Hilten BJ van der Helm FC Muscle weakness and lack of reflex gain adaptation predominate during post-stroke posture control of the wrist. J Neuroeng Rehabil (2009) 6:29. 10.1186/1743-0003-6-29 19627607 PMC2732629

[32] Lee SW Triandafilou K Lock BA Kamper DG. Impairment in task-specific modulation of muscle coordination correlates with the severity of hand impairment following stroke. PLoS One (2013) 8(7):e68745. 10.1371/journal.pone.0068745 23874745 PMC3712930

[33] Zehr EP Carroll TJ Chua R Collins DF Frigon A Haridas C Possible contributions of CPG activity to the control of rhythmic human arm movement. Can J Physiol Pharmacol (2004) 82(8–9):556–68. 10.1139/y04-056 15523513

[34] Lang CE Macdonald JR Reisman DS Boyd L Jacobson Kimberley T Schindler-Ivens SM Observation of amounts of movement practice provided during stroke rehabilitation. Arch Phys Med Rehabil (2009) 90(10):1692–8. 10.1016/j.apmr.2009.04.005 19801058 PMC3008558

[35] Cole KJ Abbs JH. Coordination of three-joint digit movements for rapid finger-thumb grasp. J Neurophysiol (1986) 55(6):1407–23. 10.1152/jn.1986.55.6.1407 3734863

[36] Yokogawa R Hara K. Manipulabilities of the index finger and thumb in three tip-pinch postures. J Biomech Eng (2004) 126(2):212–9. 10.1115/1.1691444 15179851

[37] Santello M Flanders M Soechting JF. Postural hand synergies for tool use. J Neurosci (1998) 18(23):10105–15. 9822764 10.1523/JNEUROSCI.18-23-10105.1998 PMC6793309

[38] Mason CR Gomez JE Ebner TJ. Hand synergies during reach-to-grasp. J Neurophysiol (2001) 86(6):2896–910. 10.1152/jn.2001.86.6.2896 11731546

[39] Nguyen HB Lee SW Harris-Love ML Lum PS. Neural coupling between homologous muscles during bimanual tasks: effects of visual and somatosensory feedback. J Neurophysiol (2017) 117(2):655–64. 10.1152/jn.00269.2016 27852730 PMC5288480

[40] Gomez-Rodriguez M Peters J Hill J Schölkopf B Gharabaghi A Grosse-Wentrup M. Closing the sensorimotor loop: haptic feedback facilitates decoding of motor imagery. J Neural Eng (2011) 8(3):036005. 10.1088/1741-2560/8/3/036005 21474878

[41] Squeri V Masia L Taverna L Morasso P. Improving the ROM of wrist movements in stroke patients by means of a haptic wrist robot. Conf Proc IEEE Eng Med Biol Soc (2011) 2011:2077–80. 10.1109/IEMBS.2011.6090385 22254746

[42] Squeri V Casadio M Vergaro E Giannoni P Morasso P Sanguineti V. Bilateral robot therapy based on haptics and reinforcement learning: feasibility study of a new concept for treatment of patients after stroke. J Rehabil Med (2009) 41(12):961–5. 10.2340/16501977-0400 19841824

[43] Kofler M Fuhr P Leis AA Glocker FX Kronenberg MF Wissel J Modulation of upper extremity motor evoked potentials by cutaneous afferents in humans. Clin Neurophysiol (2001) 112(6):1053–63. 10.1016/S1388-2457(01)00540-5 11377265

[44] Jones CL Cable Actuated Finger Exoskeleton Development and Examination of Index Finger and Thumb Coupling [Ph.D. Dissertation]. Chicago, IL: Illinois Institute of Technology (2014).

